# Role of Deep Learning in Loop Closure Detection for Visual and Lidar SLAM: A Survey

**DOI:** 10.3390/s21041243

**Published:** 2021-02-10

**Authors:** Saba Arshad, Gon-Woo Kim

**Affiliations:** Intelligent Robots Laboratory, Department of Control and Robot Engineering, Chungbuk National University, Cheongju-si 28644, Korea; sabarshad1000@gmail.com

**Keywords:** simultaneous localization and mapping, loop closure detection, deep learning, neural networks, autonomous mobile robots

## Abstract

Loop closure detection is of vital importance in the process of simultaneous localization and mapping (SLAM), as it helps to reduce the cumulative error of the robot’s estimated pose and generate a consistent global map. Many variations of this problem have been considered in the past and the existing methods differ in the acquisition approach of query and reference views, the choice of scene representation, and associated matching strategy. Contributions of this survey are many-fold. It provides a thorough study of existing literature on loop closure detection algorithms for visual and Lidar SLAM and discusses their insight along with their limitations. It presents a taxonomy of state-of-the-art deep learning-based loop detection algorithms with detailed comparison metrics. Also, the major challenges of conventional approaches are identified. Based on those challenges, deep learning-based methods were reviewed where the identified challenges are tackled focusing on the methods providing long-term autonomy in various conditions such as changing weather, light, seasons, viewpoint, and occlusion due to the presence of mobile objects. Furthermore, open challenges and future directions were also discussed.

## 1. Introduction

Over the past few decades, simultaneous localization and mapping (SLAM) has been one of the most actively studied problems in autonomous robotic systems. Its main function is to enable the robot to navigate autonomously in an unknown environment by generating the map and accurately localize itself in the map. During localization, the robot must correctly recognize the previously visited places known as true loops. This recognition is done by one of the components of SLAM, known as loop closure detection. A true-loop closure detection helps the SLAM system to relocalize and enhances the mapping accuracy by reducing the accumulated drift in the map due to robot motion [1]. However, the existing loop closure detection systems are not yet that efficient to accurately detect the true loops. The parameters affecting the detection of true loops are changing illumination, environmental conditions, seasons, different viewpoints, occlusion in features due to the presence of mobile objects, and the presence of similar objects in different places.

Earlier studies have used point features for the detection of closed loops. Point features, such as scale invariant feature transform (SIFT) [2] and speedup robust features (SURF) [3], etc., used for visual loop closure detection are computationally expensive and are not suitable for real-time visual SLAM systems [4,5,6]. To enhance computational efficiency, many researchers have developed the bag-of-words (BoW)-based loop closure detection methods [7,8,9,10]. These methods store the visual information of the environment as a visual dictionary and generate the clusters where each cluster represents a “word”. The computational efficiency of BoW-based loop detection methods is further boosted with the inverted-index approach used for the data retrieval of previously visited places [10]. Though the BoW approach provides a fast solution to the handcrafted features-based loop closure detection, it requires a large amount of memory to store the visual words [11]. Most of the BoW-based loop detection algorithms generate fixed-sized vocabulary in an offline step and perform the loop closure detection in an online step to further reduce the computational cost [5,10]. Such methods perform well only if the loop closure detection is performed in the preknown environment and are not practical for unexplored environments. To address this issue, many researchers have created the BoW vocabulary in an online step to enable the system to work in real environments [10]. However, these methods are still inefficient as the memory requirement increases with increased vocabulary size. The recent studies using convolution neural network (CNN) features for loop closure detection have proven to be more robust against the above-mentioned challenges [12,13,14,15]. In addition, efforts have been made to reduce the memory usage of stored features through deep neural networks [16]. However, detecting truly closed loops is still an open problem.

Loop closure detection is of key importance to the SLAM system for the relocalization of a robot in a map. Several survey articles have extensively discussed the SLAM algorithms and their efforts to improve closed-loop detection. Hence, a thorough taxonomy is required to categorize the loop closure detection algorithms. In recent research [17], an extensive comparative analysis for feature-based visual SLAM algorithms was presented. The existing research is grouped into four categories based on visual features, i.e., low level, middle level, high level, and hybrid features, and highlighted their limitations. Another review for SLAM systems is provided where the scope is only limited to the vision-based SLAM algorithms [18]. Similarly, Sualeh et al. [19] developed a taxonomy of SLAM algorithms proposed in the last decade and discussed the impact of deep learning to overcome the challenges of SLAM. The loop closure detection was not thoroughly highlighted. A detailed survey of deep learning-based localization and mapping is provided in [20]. Other surveys and tutorials focusing on the individual flavors of SLAM include the probabilistic formulation of SLAM [21], pose-graph SLAM [22], visual odometry [23], and SLAM in dynamic environments [24]. We recommend these surveys and tutorials to our readers for a detailed understanding of SLAM. 

In this survey, state-of-the-art loop closure detection algorithms developed for visual and Lidar SLAM in the past decade have been discussed and categorized into three major categories of vision-based, Lidar-based, and deep learning-based loop closure detection methods. To the best of our knowledge, this is the first survey article that provides an extensive study primarily focused on loop closure detection algorithms for SLAM. Moreover, open challenges are also identified.

The rest of the paper is organized as follows: In Section 2, the existing research of vision and Lidar-based loop detection using conventional approaches is discussed along with their limitations. Section 3 explains the state-of-the-art deep learning-based loop closure detection methods. Section 4 summarizes the challenges of conventional loop closure detection schemes and the existing research on deep learning-based loop detection to overcome these challenges. Finally, Section 5 concludes the survey.

## 2. Taxonomy of Loop Closure Detection 

One of the essential parts of SLAM is the recognition of the previously mapped places and eliminating the incremental drift by recognizing the premapped environment. For loop closure detection, the estimation process cannot be trusted because of inconsistency. Thus, a dedicated algorithm is needed for the relocalization of the vehicle in a prebuilt map. In this section, the loop closure detection techniques are grouped into two major categories: vision-based and Lidar-based loop closure detection. Each category further groups the existing research on the basis of data acquisition and matching methods.

### 2.1. Vision-Based Loop Closure Detection

As imagery provides rich visual information, most of the methods make use of camera sensors for loop detection. Based on the matching schemes, we have grouped the vison-based loop detection methods as image-to-image matching [25], map-to-map matching [26], and image-to-map matching [27] schemes, as done in [28].

#### 2.1.1. Image-to-Image Matching

Loop closure detection methods performing the image-to-image matching using the correspondence between the visual features are grouped in this category. These methods do not require the metric information of the features; instead, they apply topological information.

Bag-of-words [29] model has been widely used for loop closure detection. It first generates a vocabulary consisting of visual words where each word is a combination of some features extracted from a large training dataset. These features are clustered using the K-means clustering algorithm [30], as it is effective for unsupervised learning [31]. While searching for a similar match for the current image, the BoW method converts the image into a set of descriptors and for each descriptor. It searches for the closest cluster center to generate a BoW vector which is used for image matching with previously seen images. The vocabulary generation process is done as a preprocessing step either offline or online.

##### Methods with Offline Vocabulary

This category includes the loop detecting techniques using the bag-of-words approach where image features are discretized in the descriptor space and a unique vocabulary word is assigned to the group of similar visual or binary features. This vocabulary is generated in a hierarchical structure which enhances the matching performance.

In [8], a probabilistic framework is presented, FABMAP, for appearance-based place recognition and loop closing. FABMAP does not only detect the previously visited places on the map but also identifies the new places and augments the map. The algorithm applies the Chow–Liu tree [32] for building a generative model of visual BoW vocabulary with 11,000 words. These visual words consist of groups of visual features extracted from the images using a SURF detector/descriptor [33]. The system has been evaluated in an outdoor environment dataset. The complexity of FABMAP is linear to the number of places on the map. Although FABMAP achieved high performance, it was suitable for a few kilometers of trajectory. Also, the system performance degraded in the environments other than the training data. As FABMAP is using SURF features, it requires 400 milliseconds for only the feature extraction process.

To enhance the applicability of the FABMAP in large-scale environments, FABMAP 2.0 [34] was proposed for the 70 km and 1000 km trajectories dataset. It has also applied the inverted index with the BoW model for place recognition, generating a vocabulary of 100,000 words which improves the overall performance of the system in terms of loop detection and resource consumption. It has become the gold standard for loop detection, but the robustness decreases if the similar structure appears in the images for a long time [35].

Vocabulary generated using visual features such as SIFT and SURF provides high performance because of invariance to light, scale, and rotation. However, these features required a longer computation time [35,36,37,38]. This problem was addressed by the usage of binary features such as BRIEF [39], BRISK [40], and ORB [41]. As their information is compact so they are fast to compute and compare thus allowing much faster place matching. For the first time, binary features have been used in [9,42], Fast detector and BRIEF descriptor, for building a vocabulary of binary words. The system can perform the loop detection and verification at one order of magnitude less than the other similar techniques. As the BRIEF is not invariant to significant scale and rotation, these methods are good for loop detection with planar camera motion.

The BoW-based loop closure detection methods depend on the appearance features and their existence in the dictionary, ignoring the geometric information and relative position in the space, thereby resulting in false loops due to similar features appearing in different places [43]. Also, in the presence of dynamic objects, the similarity in the loop scene is reduced, thus causing the system to lose stability.

##### Methods with Online Vocabulary

The above-mentioned schemes build the vocabulary in offline steps. The systems trained on the prebuild vocabulary show high performance on the same dataset, but the performance degrades if the traversed places are inconsistent with the trained dataset. This problem is addressed by the generation of the vocabulary in an online step.

Offline vocabulary is not suitable for the dynamic robot environment. In [44] a loop closure detection method for a dynamic indoor and outdoor environment is developed by incrementally generating and updating the vocabulary, in an online step, through feature tracking among consecutive frames. The loop candidates are identified by a likelihood function that is based on inverse frequency of corresponding image features. After the likelihood evaluation, the vocabulary is updated based on new features extracted from the current image. Through extensive experiments, it is shown that the incremental vocabulary generation achieves a higher number of true positives in comparison to [37].

The BoW provides fast and easy loop detection. However, the performance of such systems is highly dependent on the appearance of a place. Thus, they suffer from a perceptual aliasing problem, i.e., occurrence of similar features in different places or drastic change in appearance of a place due to variation in environmental conditions. Kejriwal et al. [45] generated a bag-of-word-pairs dictionary using quantized SURF features by incorporating the spatial co-occurrence information of the image features to improve the recall rate and reported better performance than [44].

To deal with the occlusion due to the presence of dynamic objects, SIFT features have been used to enhance the loop detection accuracy for monocular SLAM [46]. The appearance changes in the dynamic environment due to moving objects have been detected through features projection from keyframes to current image and comparison among them. Tracking is performed through matching features. As a result, image comparisons and dynamic change in the environment are detected through gradual change in image portions. As SIFT features are computationally expensive, the system ensures the real-time performance through GPU acceleration and multithread programming. Similarly, in [47], SURF and BRIEF features have been extracted to perform the word training for loop detection in long-term autonomous driving. To improve the detection accuracy of BoW-based closed-loop detection in a dynamic environment, Xu et al. [48] performed the discrimination among feature points that belong to the static and dynamic objects. The algorithm first detects and removes the feature points belonging to the dynamic objects and then generates the BoW vocabulary using the static features.

#### 2.1.2. Map-to-Map Matching

Methods performing map-to-map feature matching detect the loops by using the visual features and relative distance between features common to two submaps. In [26], loop closure detection is performed in monocular SLAM by using the geometric compatibility branch and bound (GCBB) algorithm which matches the submaps based on the similarity in visual features common in both submaps and their relative geometry. However, the system is not suitable for sparse maps [28]. The major limitation of such methods is that the maps are either too sparse to be distinctive or too complex such that they cannot be completely explored for high performance in real-time. For such methods, the exploration space can be reduced by using the position information of the map features as done in [49].

#### 2.1.3. Image-to-Map Matching

This group includes the loop detection methods which use the correspondences between the visual features of the current camera image and the feature map. While in image-to-map matching, the aim is to determine the camera pose relative to the point features in the map and matching is based on appearance features along with their structure information.

In [25], a feature-tracking and loop closing method is proposed for monocular SLAM using appearance and structure information. At each time step, 16D SIFT features have been extracted from the current image representing the appearance information and matching with the map features using BoW model. The BoW appearance model helps to identify the part of the map that is similar to the current image by comparing image features with the map features and generating the loop closure candidates. The map is stored as a graph where each node stores the structure of landmarks. The structure for loop candidates is matched through landmark appearance models. The current pose relative to the map features is further determined by MLESAC [50] and the three-point pose algorithm [51].

William et al. [52] proposed a method for camera-pose estimation relative to the map for relocalization and loop closing through an image-to-map matching scheme. The feature map is built using the visual and metric information of landmarks. The appearance information of map features is learned using a randomized tree classifier [53], and correspondences between the current image and map are generated by landmark recognition. Once the landmarks are recognized in an image, the camera pose is determined using estimated metric information. For this purpose, a global metric map is divided into submaps. The relative positions of these submaps are determined by the mutual landmarks. The global map is represented by a graph where each node is a submap and edges between the nodes represent the transformation between submaps. Tracking is performed between the current and the previous submap, thus merging the maps. In the case of true overlap, the relative transformation between submaps is determined by the poses from their trajectories and an edge is added between the two consecutive submaps, thus representing a detected loop. Though it performs well in relocalization and loop closing, the randomized list classifier is memory inefficient.

Xiang et al. [54] proposed direct sparse odometry with loop closure detection (LDSO) as an extension of direct sparse odometry (DSO) [55] for monocular visual SLAM. The DSO ensures the robustness of the system in a featureless environment. To retain the repeatability of the feature points, LDSO extracts ORB features from keyframes. The loop closure candidates are selected using the BoW approach as used in [9]. Later, the RANSAC PnP [56] is applied for the verification of loop candidates. Raul et al. [57] addressed the relocalization and loop closure problem in keyframe-based SLAM by using the ORB feature. The proposed solution applies the image-to-map feature matching scheme and is robust to scale changes from 0.5 to 2.5 and 50 degrees of viewpoint changes. The loop can be detected and corrected at a 39 milliseconds frame rate in the database of 10,000 images. Due to scale and viewpoint invariance, the proposed method achieves a higher recall rate in comparison to [8,9,34].

### 2.2. Lidar-Based Loop Closure Detection

Vision-based loop closure detection for a long-term autonomous system is a challenging task due to large viewpoint and appearance changes. When such systems revisit a place, they are subject to extreme variations in seasons, weather, illumination, and a viewpoint along with the dynamic objects. These environmental changes make robust place recognition extremely difficult. These limitations can be handled by the LiDAR, up to some extent, as Lidar measurements are less prone to light and environmental changes in comparison to vision sensors, providing a 360-degree field of view. Unlike vision-based loop detection, research for Lidar-based solutions is rare. One of the reasons could be the high cost of LiDAR sensors which prevents the wider use. Another reason is that the LiDAR point clouds only contain the geometry information while images contain rich information; thus the place recognition is a challenging problem when using point clouds. The existing research on Lidar-based loop detection can be generally grouped into histograms and segmentation-based methods.

#### 2.2.1. Histograms

Histogram extracts the feature values of points and encodes them as descriptors using global features [58,59,60] or selected keypoints [61,62,63,64]. One of the approaches used by these methods is the normal distribution transform (NDT) histogram [65], [66] which provides the compact representation of point cloud maps into a set of normal distributions. In [67], an NDT histogram is used to extract the structural information from Lidar scans by spatially dividing the scans into overlapping cells. NDT is computed for each cell and instances of certain classes of NDT in range intervals constitute the histograms. The authors have compared the histograms using the Euclidean distance metric [68]. It is shown that structural information provided by the histograms of NDT descriptors improves the accuracy of the loop detection algorithm. A similar approach is used in [69] for loop closure detection where scan matching is performed using the histograms of NDT descriptors.

NDT histogram-based methods are computationally expensive. To overcome the computational overhead, many researchers have put efforts into developing fast loop detection methods. In [58], the performance of the loop detection method presented in [67] has been improved and the computational cost is reduced by using the similarity measure histograms extracted from Lidar scans that are independent of NDT. Lin et al. [70] developed a fast loop closure detection system for Lidar odometry and mapping. It performs similarity matching among keyframes through 2D histograms. Another approach used for reducing the matching time is proposed in [62], where place recognition is performed by using 3D point cloud keypoints and 3D Gestalt descriptors [71,72]. The descriptors of current scan keypoints are matched with the point cloud map and a matching score is computed for each keypoints using nearest neighbor voting scheme. The true loop is determined by the obtained highest voting score after geometric verification.

The histogram-based methods can handle the two major issues: rotation invariance for large viewpoint changes and noise handling for spatial descriptors, as spatial descriptors are affected by the relative distance of an object from Lidar [61,73,74]. The major limitation of histogram-based methods is that they cannot preserve the information of the internal structure of a scene, thus making it less distinctive and causing false loop detections.

#### 2.2.2. Segmentation

The loop detection methods using a point-cloud-segmentation approach are based on shapes or objects recognition [75,76,77,78,79,80]. In such methods, segmentation is performed as a preprocessing step because a priori knowledge about location of objects, that are to be segmented during robot navigation, is needed. The segment maps provide a better representation of a scene where static objects may become dynamic and are more related to the ways human’s environment perception. One of the advantages of such techniques is the ability to compress the point cloud map into a set of distinctive features which largely reduced the matching time and likelihood of obtaining false matches. Douillard et al. [81] provide a detailed discussion on several segmentation methods for Lidar point clouds including ground segmentation, cluster-all, base-of, base-of with ground method for dense data segmentation, and Gaussian process incremental sample consensus, mesh-based segmentation for sparse data. SegMatch [82] uses the cluster-all method for point cloud segmentation and extracts two types of features including eigenvalue-based and shape histograms. The features are segmented as trees, vehicles, buildings, etc., and matching is done by using a random forest algorithm [83]. It is observed that SegMatch requires real-time odometry for loop detection and does not perform well when using only the Lidar sensor. Also, the maps generated by SegMatch are less accurate. A similar segmentation approach is used in [84] to enhance the robustness of loop closure detection by reducing the noise and resolution effect. The point cloud descriptor encodes the topological information of segmented objects. However, the performance degrades if the segmentation information is not sufficient. In recent research [85], an optimized Lidar odometry and mapping algorithm is proposed in integration with SegMatch-based loop detection [82] to enhance the robustness and optimization of the global pose. The false matches are removed using ground plane constraints based on RANSAC [86]. Tomono et al. [87] applied a coarse-to-fine approach for loop detection among feature segments to reduce the processing time where lines, planes, and balls are used for coarse estimation instead of feature points.

Based on the literature reviewed in this section, the benefits and limitations of each method are summarized in Table 1.

## 3. Role of Deep Learning in Loop Closure Detection

In the past few years, deep learning has been introduced in visual and Lidar SLAM systems to overcome the challenges of truly-closed-loop detection [13,14,15,16,19,88,89,90]. The deep learning-based loop detection methods are known to be more robust to changing environmental conditions, seasonal changes, and occlusion due to the presence of dynamic objects [91]. This subsection presents the state-of-the-art deep learning-based loop closure detection methods using camera and Lidar sensors. The main characteristics of the algorithms are tabulated in Table 2. The table represents the reference of the algorithms followed by the year of publication, the sensors used for environment perception, the type of features used for environment representation, the neural network used by the algorithm, the type of environment for which the algorithm is developed, the loop closure challenges addressed by the algorithm, i.e., variation in weather, seasons, light, and viewpoint, computational efficiency, dynamic interference in the environment due to moving objects and the semantics used for environment classification.

### 3.1. Vision-Based Loop Closing

In deep learning-based visual loop closure detection algorithms, research efforts have been made to overcome the limitations of handcrafted feature-based methods. In a recent research [89], a multiscale deep-feature fusion-based solution is presented where abstract features are extracted from AlexNet [92] pre-trained on ImageNet [93] and fused with different receptive fields to generate fixed-length image representations that are invariant to illumination changes. A similar approach is used in [94] where features are extracted from fast and lightweight CNN to improve the loop detection accuracy and computation speed.

Many researchers have used semantics-based objects and scene classification for loop detection through deep learning [95,96]. Semantic segmentation classifies each image pixel according to the available object categories. All pixels, in an image, with same object class label are grouped together and represented with same color. Maps with semantic information enable the robots to have a high-level understanding of the environment.

Another approach used by the researchers for loop detection is autoencoders. The autoencoder compresses the input frame and regenerates it to the original image at the output end of the system [88,97]. Merril et al. [98] proposed an autoencoder-based unsupervised deep neural network for visual loop detection. The illumination invariance is achieved by generating HoG descriptor [99] from the autoencoder at output instead of the original image. The network is trained on the Places dataset [100] containing images from different places, primarily build for scene recognition. During the robot navigation, there may exist similar objects in different places which can greatly affect the performance of the algorithm when it is executed for a sequence of images of a path. The major limitation of this approach is that the autoencoder cannot show which keyframe in the database matches with the current image; instead, it can only detect if the current place is already visited or not. Gao et al. [88] have used the deep features from the intermediate layer of stacked denoising autoencoder (SDA) [101] and performed the comparison of the current image with previous keyframes. This method is also time-consuming as the current image is compared to all the previous images. Also, the perceptual aliasing problem is not addressed, which may result in false loops and incorrect map estimations.

### 3.2. Lidar-Based Loop Closing

To improve the matching time and detection accuracy of histogram-based loop detection methods, Zaganidis et al. [102] generated an NDT histogram-based local descriptor using semantic information obtained from PointNet++ [103]. Here, [104] implemented PointNetVLAD [105] which integrates PointNet [106] and NetVLAD [107] to generate a global descriptor from 3D point cloud. Similarly, LocNet [108] applies a semi-handcrafted deep network to generate a global representation of scan maps for place matching and loop detection.

In deep learning-augmented segment-based loop detection methods, the SegMap [109] produces segments of the scene incrementally as the robot navigates and passes those segments to a deep neural network to generate a signature per segment. A loop is detected by matching the segment signatures. SegMap aims to extract meaningful features for global retrieval while the semantic class types were limited to vehicles, buildings, and others. The performance of SegMap is further improved in [110].

Though segmentation-based loop detection methods are successful to enhance the performance in terms of processing time, they are highly dependent on segmentation information available in the environment. One of the solutions can be the formulation of a more generic and robust descriptor using segmentation information from multiview Lidar scans.

## 4. Challenges of Loop Closure Detection and Role of Deep Learning

Based on the existing literature and their limitations presented in Section 3, the major challenges of loop closure detection methods are identified. This section lists the major challenges and role of deep learning in SLAM systems to overcome those challenges.

### 4.1. Perceptual Aliasing

**Figure 1:**
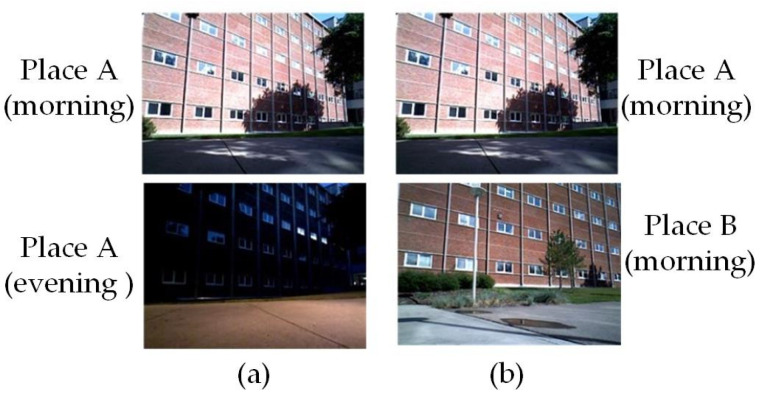
(**a**) Same place with different appearance and (**b**) similar looking different places.

In an environment, the objects may have some visual, geometric, and topological features based on appearance, structure, and relative position of objects as depicted in Figure 1. Similar features may appear in different places such as in many buildings that have the same structure, color, and topological features, in corridors in a building with the same structure, or in doors that have the same geometry. This occurrence of similar features at different locations causes the loop detection algorithm to generate false loop correspondences and is termed as a “perceptual aliasing” problem. Perceptual aliasing is one of the main reasons for the failure of appearance-based loop detection methods. Many BoW-based methods generate false correspondences as they only consider similar visual features for true-loop detection [88]. This problem is well addressed in recent work by combining multiview information of a place, instead of single view, through deep neural networks [121]. Also, the temporal information is embedded in the descriptors by concatenating the descriptors of consecutive frames. Through experiments, it is shown that image descriptors generated from the sequence of images are more robust and distinctive in comparison to the descriptors generated from single image.

### 4.2. Variation in Environmental Conditions

**Figure 2:**
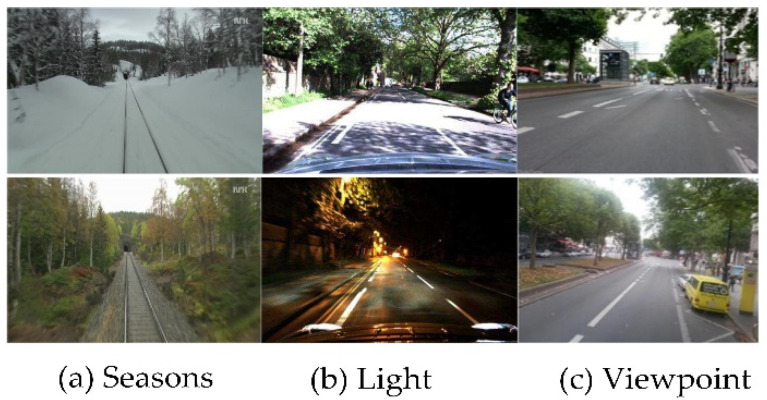
Change in appearance of places due to variation in (**a**) seasons [125], (**b**) light [124], and (**c**) viewpoint [126].

The loop closure is an open problem due to variation in illumination conditions [124], seasons [125], and viewpoints [126]. Figure 2 depicts the variation in seasons from summer to winter, light changes from day to night, and viewpoint variation due to lateral and angular changes.

For robot navigation in environments with viewpoint variations, the conventional approaches succeed up to some extent to achieve high performance in loop closure detection. In the case of light, weather, and seasonal variations, the features cannot be detected i.e., features detected during daytime are not detectable at night due to light effects. Seasonal changes affect the appearance of the environment drastically, e.g., leaves disappear in autumn, the ground is covered with snow in winter, etc. Similarly, weather conditions such as rain, clouds, and sunlight change the appearance of the environment. It is complicated to overcome these challenges using conventional methods as they are sensitive to such environmental conditions [88].

To overcome the challenges of changing environmental conditions, many researchers have proposed CNN-based loop detection methods that are robust to the variance of illumination and other conditions [16,88,98,122]. In [98], an unsupervised deep neural network is used to achieve illumination invariance in visual loop detection. Autoencoder-based loop detection methods using unsupervised deep neural networks achieved state-of-the-art performance for loop detection in variable environmental conditions [88,127]. However, these methods are not scale invariant. The viewpoint invariance problem up to 180-degree rotation change is addressed in [84] through an object-based point-cloud-segmentation approach.

BoW features are sensitive to illumination changes and cause loop detection failure in severe environmental condition changes. Chen et al. [89] extracted the abstract features from AlexNet and improved the illumination invariance through multiscale deep-feature fusion. For varying weather conditions, [118] performed camera-LiDAR-based loop closure detection using a deep neural network.

### 4.3. Dynamic Environment

**Figure 3:**
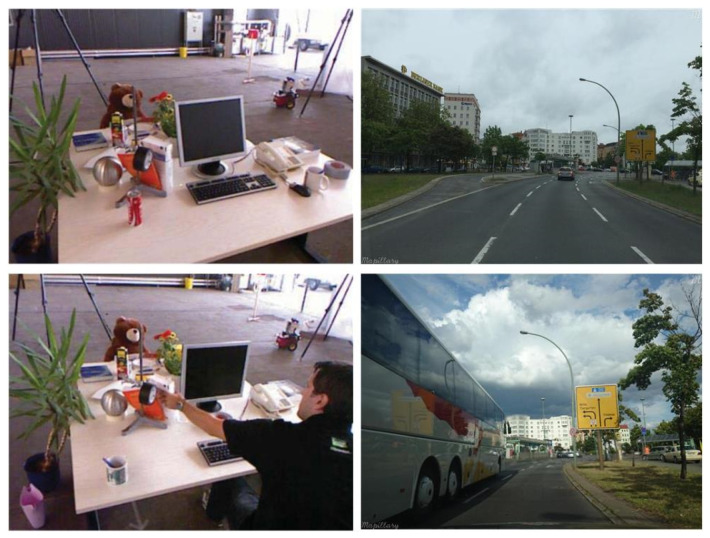
Occlusion in features of same place due to moving objects such as person [128] and vehicles [126].

The presence of moving objects in the environment is one of the major challenges for true loop detection. The mobile objects cause occlusion to the essential features in the scene [126,128], as shown in Figure 3; thus, the available features are not sufficient for the algorithm to detect the loop closure, leading to the closed-loop detection failure.

Many researchers have addressed this problem to improve the accuracy of the algorithm. The BoW-based loop closure detection methods perform well in a static environment. However, the detection performance decreases in the presence of dynamic objects due to reduced feature similarity in loop scenes. This issue can be better addressed using the semantic information of the environment as the semantic extraction is not affected if there are mobile objects or people [112].

Hu et al. [95] fused the object-level semantic information with the point features based on the BoW model [129] to enhance the image similarity in loop scenes and stabilize the system in a dynamic environment. Object-level semantic information is extracted using Faster R-CNN [130], pretrained on the COCO dataset [131]. It is shown that the point feature matching fused with semantics achieves better detection precision in comparison to only BoW-based loop detection. In [96], the object-based semantic information is embedded with the ORB features to improve the performance of the overall SLAM framework. The proposed framework extracts the semantic information of objects and assigns the class labels to the feature vectors which lie within the boundary boxes. Feature matching is only performed among the features with same class labels. Thus, avoiding the wrong matches and reducing the computation time for the loop closure detection thread. Memon et al. [16] combined the supervised and unsupervised deep learning methods to speed up the loop detection process. The true-loop detection is ensured by removing the features from dynamic objects that are either moving or temporarily static. Through deep learning, the proposed system performs eight times faster loop closure detection at low memory usage in comparison to traditional BoW-based methods.

### 4.4. Real-Time Loop Detection

In SLAM, the mapping and loop detection run in parallel threads. As the robot keeps on generating the environment map along the trajectory, the loop detection algorithm compares the current frame (in visual SLAM) with all previously seen images to detect the closed loop. As the map size increases, the similarity computation time for each frame increases which slows down the system and is not suitable for real-time applications [88]. Many approaches have been proposed in the past to overcome this challenge such as selecting random frames [132,133] or the fixed keyframes as used in ORB-SLAM2 [134] for comparison with the current frame, but still, the system will slow down in case of longer trajectories and also the probability of detecting true loop will decrease [88]. Thus, developing a real-time loop closure detection algorithm able to optimize the computation time with the variable map size is one of the major challenges. In the previous few years, deep learning-based loop closure detection methods have been developed to enhance the computational efficiency through different schemes such as reducing the descriptor size [121], reducing the deep network layers during deployment [94,98], matching features of same semantic class [96], generalizing scene representation with segmentation and matching segment feature descriptors instead of point features [109].

## 5. Conclusions and Future Research Directions

SLAM is an integral part of most autonomous robots. This article presents an extensive survey primarily focused on loop closure detection methods based on visual and Lidar features and groups them into two major categories. Based on the limitations of each approach, the major challenges of loop closure detection are identified. The survey also argues on how those challenges are addressed by the deep learning-based methods. From the reviewed literature, it is observed that loop detection methods based on deep neural networks proved to be robust to the challenges, but true-loop detection is still an open issue as both the camera and Lidar-based deep-learning loop closure detection approaches have some limitations.

The vision-based loop closure detection methods are sensitive to illumination variations and cannot work, but LiDAR can. Similarly, the Lidar-based methods fail in weather changes such as rain, while vision-based methods can perform comparatively well [135]. Thus, there is a need for research in visual–LiDAR fusion-based loop closure detection to take advantage of both modalities for achieving robustness against illumination and environmental changes [118]. To this end, the LiDAR scan analysis for feature detection and camera–LiDAR calibration are the primary problems to be addressed [118]. The semantics provides high-level understanding of the environment allowing the robot to percept the environment like the humans. One of the major limitations of semantics-based loop detection methods is the assumption that there are enough objects learned by the pretrained CNN model. In a real environment, this assumption may not be satisfied. Also, learning-based methods are computationally expensive, and the performance is dependent on the dataset used for training the network.

## Figures and Tables

**Table 1:** Summary of the benefits and limitations of camera and Lidar-based loop closure detection methods.

Method			Benefits	Limitations
Vision-based	Image-to-Image	Offline Vocabulary	Does not require the metric information of the features Dependent on the appearance features and their existence in dictionary Good for loop detection with planar camera motion	Not suitable for dynamic robot environment Memory consumption is proportional to vocabulary size Performance reduces if tested on different dataset.
		Online Vocabulary	Allows to learn features in real time	Memory consumption is proportional to vocabulary size Does not use geometric information
	Map-to-Map		Detects true loops when common features exist in two submaps	Not suitable for sparse maps. Cannot achieve high performance for complex dense maps.
	Image-to-Map		High performance when tuned for 100% precision Allows online map feature training for real environment	Memory inefficient
Lidar-based	Histograms		Provides rotation invariance for large viewpoint changes Noise handling for spatial descriptors	Cannot preserve distinctive information of internal structure of a scene
	Segmentation		Compresses large point cloud maps into set of distinctive features Reduced matching time	Requires prior knowledge of object locations

**Table 2:** State-of-the-art deep learning-based loop closure detection methods for visual and Lidar SLAM.

Ref.	Year	Sensor	Components	Deep Learning Algorithm	Env	Challenges						Seman-tics
						Weather	Seasons	Light	Viewpoint	Effi-ciency	Dynamic Env	
[89]	2019	C	CNN feature	AlexNet	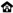 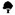	-	-	+	-	-	-	-
[95]	2019	C	SIFT, SURF, ORB	Faster R-CNN	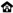	-	-	-	+	-	+	+
[96]	2018	C	ORB	Yolo [111]	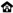 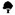	-	-	+	+	+	+	+
[98]	2018	C	HoG	Autoencoder	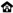 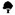	+	+	+	+	-	+	-
[102]	2019	L	Semantic-NDT	PointNet++ [103]	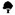	-	-	+	+	+	+	+
[108]	2018	L	Semi-handcrafted	Siamese	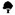	-	-	+	+	+	+	-
[109]	2018	L	SegMap	CNN	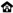 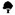	-	-	-	+	-	-	-
[110]	2020	L	SegMap	CNN	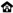 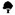	-	-	-		+	+	-
[112]	2016	C	SIFT, SURF, ORB	PCANet [113]	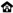 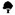	-	-	+	+	-	-	-
[114]	2020	L	Semantic class	RangeNet++ [115]	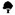	-	-	-	+	-	+	+
[116]	2020	C	CNN feature	ResNet18 [117]	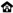 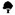	+	+	+	+	+	+	-
[118]	2020	C/L	CNN feature	VGG16 [119]	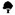	+	-	+	-	-	+	-
[120]	2018	C	CNN feature	VGG16	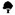	+	+	+	+	-	-	-
[121]	2019	C	CNN Multiview descriptor	ResNet-50 [117]	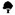	+	+	+	+	-	+	-
[122]	2019	C	Semantic feature	Hybrid [123]	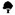	+	+	+	+	-	-	+

L: LiDAR; C: Camera; 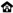: Indoor; 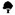: Outdoor; +: Present; -: Absent.

## Data Availability

Not Applicable.
